# Experimental Researches Applied to Physiology and Pathology

**Published:** 1853-04

**Authors:** E. Brown-Séquard

**Affiliations:** Paris


### MEDICAL EXAMINER,

AND

### RECORD OF MEDICAL SCIENCE.

NEW SERIES.—NO. C.—APRIL, 1853.

### ORIGINAL COMMUNICATIONS.

### *Experimental Researches applied to Physiology and Pathology.*

By E. Brown-Séquard, M. D., of Paris.

(Continued.)

#### XXV.—ON THE TREATMENT OF EPILEPSY.

I have made numerous experiments with regard to the treatment of this dreadful affection, and I intend to publish them, in extenso, when some points that are still obscure, have become clear to my mind. Here I will merely relate some of the most important results of my researches. As I have had the opportunity during the last three or four years of observing every day a great many animals (more than a hundred) which had a convulsive affection resembling epilepsy very much, I have been able to discover some very interesting facts, among which are the following:

1st. For each epileptic animal, the number of fits, in a given time, is generally in a direct proportion with the quantity of food taken.

2d. There is an inverse proportion between the amount of exercise and the number of fits.

3d. Cauterisation of the mucous membrane of the larynx is able either to cure or to relieve these epileptic animals.

The convulsive affection existing in almost all these animals was the consequence of a transversal section of a lateral half of the spinal cord, in the dorsal or in the lumbar region.

I have already published the results of my experiments on epilepsy, in my lectures before large classes of Physicians and Medical students, both in France in 1851 and in this country in 1852.

These results are in perfect accordance with the views of Dr. Marshall Hall in relation to epilepsy. As the views of this eminent biologist are generally known, I need not expose them, and I will merely remind my readers of the three following points:

1st. The first muscles that contract spasmodically in almost all, if not in all, the cases of epileptic fits, are those of the larynx and the neck: 2d, spasm of the glottis taking place then, produces suffocation, in consequence of which convulsions are produced in the trunk and the limbs; 3d, tracheotomy may prevent these convulsions by preventing suffocation, and it is known that in some cases tracheotomy has cured epilepsy.

It has been objected to Marshall Hall that in cases of poisoning by strychnine, convulsions take place even when a tracheal tube renders respiration perfectly free. This objection has no value, because the state of the spinal cord in epileptics is not the same as in men or animals poisoned by strychnine. Certainly the excitability of the spinal cord is greater in epileptics than in healthy persons, but the degree of excitability of that nervous centre is much greater in persons poisoned by strychnine than in epileptics; and, therefore, it is easy to understand that certain excitations are able to produce general convulsions in one case and not in the other.

If we give a very slight dose of strychnine to an animal, so as not to poison it, but merely to increase slightly the excitability of the spinal cord, there are no convulsions when we touch or pinch or burn the skin, but if we prevent breathing for a few seconds only, general convulsions take place, exactly as in epileptic men or animals.

It has been said also, in opposition to Marshall Hall, that a spasm of the glottis of the severest kind occurs in cases of hooping cough, of spasmodic croup and even of apoplexy, without the occurrence of any other convulsions. The answer to this objection is, that in epilepsy the spinal cord is more excitable than in these other diseases, so that the same kind of excitation does not produce the same effects.

A great many facts, that I will publish elsewhere, prove that black blood, very probably by its carbonic acid, is an excitant of the spinal cord and of the medulla oblongata. When, as is the case in asphyxia, the blood is not oxygenated and deprived of the carbonic acid constantly produced in it, or received by it from different tissues, then the excitation made on these nervous centres becomes so powerful that convulsions are produced. This is found in men and animals, even in perfect health. If the asphyxia is incomplete, convulsions are not produced, unless the excitability of the spinal cord is greater than usual, and this is the case in epileptics.

In November, 1851, at the *Ecole Pratique*, of Paris, I published for the first time, before a class of about forty young Physicians and Medical students, the results of my experiments as regards the cauterization of the larynx in epilepsy. About eight months after, Dr. Eben Watson published a paper* in which he says: "The treatment I would now propose instead of tracheotomy is simply the application of a solution of nitrate of silver, varying in strength with the requirements of the case, to the glottis of the patient, with the view of diminishing the nervous excitability of the part in question. A similar treatment has been found by me remarkably successful in alleviating and removing, in a short time, the susceptibility of the patient to laryngysmus, in cases of hooping cough, and of spasmodic croup (*laryngysmus stridulus*,) nor can I see any reason why a similar result should not ensue in chronic cases of epilepsy."

The reasons given by Dr. E. Watson are partly the same by which I had been led long before him to perform the operation he suggests. But I had also some other reasons. It is perfectly known, in the actual state of Medical Science, that the greatest changes may be produced in the nervous centres, as well as in the nerves, by a very strong excitation of the termination of the nervous fibres in the skin or the mucous membranes. On this principle are founded many modes of treatment of some dis-

* Remarks on Dr. M. Hall's theory of the relation of Laryngysmus to Epilepsy. In London Journal of Medicine, July, 1852, pp. 641–43.

eases of the spinal cord and of neuralgia. The application of caustics, blisters, cupping, hot iron, etc., is based on this principle. In accordance with it I am inclined to believe that epilepsy might be cured by a mere application of a hot iron to the skin of the neck; at least I have had two guinea-pigs cured after such an application, repeated three or four times.

The operation of tracheotomy proposed by Marshall Hall has proved successful in some cases. But it is a dangerous operation, and if it is proved that another one much slighter can produce the same good effects, it ought not to be practised.

That other operation is the cauterization of the larynx; it prevents the closure of the glottis, and thus is able to cure or to relieve epileptic patients as well as it cures some other diseases. Every learned physician knows that it is sufficient to cauterize the larynx once or twice to cure hooping cough in almost every case.

When the cause of the epileptic fits is excessive, and when the spinal cord is very excitable, to allow free breathing merely will not be sufficient to prevent the general convulsions. But their violence, if respiration is free, will be deprived of all the effect that would be produced by the excitation of black blood if breathing did not take place.

The distinction made between organic and inorganic epilepsy has not the importance that some writers seem to admit. There are alterations in the nervous system in both cases, and the only difference is that these alterations can be easily seen with the naked eye in one case and not in the other. I ought to point out that the cases of epilepsy in animals, which I have cured, were cases of organic epilepsy. These animals have been cured, although the apparent and primitive cause of the disease, *i. e.* a section of a lateral half of the spinal cord, continued to exist.

The cauterisation of the larynx on these animals was made every day, or every other day, and sometimes during two or three months. In some cases, the relief having been immediate, the cauterisation was made only twice a week. One of the animals experimented on was cured after three or four cauterisations; but the number of cauterisations necessary has been generally very much greater. When I left France in February, 1852, I had cured about a third of the animals treated by this method; and all the others, except two or three, had been very much relieved, and certainly many of them would have been cured if the treatment had been prolonged.

I knew that an animal was cured, not only by the absence of spontaneous fits, but when I could not produce a fit by giving great pain. I had found that on any epileptic animal, except immediately after a paroxysm, I could very easily produce a fit by exciting pain and more particularly by pinching or burning the skin of the face or neck. So that I am authorised to believe that when a fit was not produced by pinching or burning the face, it was because epilepsy had ceased to exist.

Some Physicians in this country have already tried on man the mode of treatment that I have found so successful on animals. From what I know of the results of their attempt, it seems to me that man is like animals in this respect. There has not been yet a complete curation: but, except in one case, there has been a very considerable diminution in the frequency and the intensity of the fits.

As Physicians who have to treat epileptics, have not to make experiments, but to cure by making use of all the best means together, I think that the treatment of epilepsy ought not to consist merely of the cauterisation of the larynx. The plan of treatment I should suggest is the following:

1st. A cauterisation of the larynx with a strong solution of nitrate of silver, (at least 60 grains to the ounce,) every day, for at least five or six weeks.

2d. A cauterisation of the skin of the neck over the spine, with a hot iron, once a fortnight, for about two or three months.

3d. Exercise and gymnastics.

4th. Make use of oxide of zinc or ammoniated copper, remedies which a very respectable Physician of Geneva, (Switzerland,) Dr. Herpin, has found successful in many cases, when their dose has been considerable.*

5th. If in a fit of epilepsy, the suffocation is very considerable; the operation of tracheotomy ought then to be performed immediately.

* See his admirable work: *Du Pronostic et du Traitement de l'Epilepsie.* Paris, 1852. Ouvrage couronné par l'Institut de France.

### XXVI. CURE OF EPILEPSY BY SECTION OF A NERVE.

It is a well known fact that epilepsy may be produced by injury to a nerve. Dr. John Cooke, in his Treatise on Nervous Diseases, says on this subject: "From the writings of Forestus, Van Swieten and Tissot, it appears that injury done to the nerves, or that a morbid state of them, has in many instances given occasion to epilepsy. In the *Edinburgh Medical Essays and Observ.*, a case is related of a violent epilepsy, which frequently occurred, which was produced by a hard cartilaginous substance, of the size of a large pea, situated upon a nerve. That this was the cause was evident, as the disease ceased on the extirpation of the tumor. In the same we have an account of epilepsy depending upon a calculus of an irregular figure, about the size of a nut, pressing on a branch of the sciatic nerve; and another in which the par vagum was compressed by a concretion of a similar kind." Darwin, in his *Zoonomia*, says, "I once saw a child about ten years old, who frequently fell down in convulsions, as she was running about in play. On examining, a wart was found on one ankle, which was ragged and inflamed, which was cut off, and the fits never recurred."

Van Swieten relates a case of curation of epilepsy by the extirpation of a hard cartilaginous body, somewhat larger than a pea, situated on a nerve of the leg.

A case is related in the *Medical and Physical Journal*, (vol. x. p. 52,) in which a cure of epilepsy was effected by the application of caustic to the nerve which accompanies the vena saphena.

Many other analogous cases are on record. Jacques Carron, (*Recueil périodique de la Soc. de Médecine de Paris*, t. xviii. p. 422,) cured a child by the extirpation of a small sebaceous tumor which existed on one of the fingers. Portal, (*Observations sur l'épilepsie*, Paris, 1827, p. 159,) cites a case observed by Fabos, in which the fits were preceded by a pain in one of the fingers. During a violent fit Fabos put a ligature around the radial nerve, and the patient was completely cured. Portal (*Anatomie Médicale*, vol. iv. p. 247,) relates that one of his pupils, Mr. Leduc, cured an epileptic by the extirpation of a hard tumor which was on one of the fingers. Joseph Frank cured by castration a patient in whom epilepsy had appeared after an injury to the scrotum. Henricus ab Heer, cited by Sennert (*Opera omnia*, vol. ii. p. 489,) having observed that during her fits, a girl used to rub her two big toes, cured her by the application of caustic to these toes. Similar cases have been related by Alexander, of Tralles, and by Wepfer.

I have cured a guinea-pig of a very violent convulsive affection, much resembling epilepsy, by a section of the sciatic nerve. This animal had been bitten by another on the toes of one of the posterior limbs. A considerable slough appeared on the wounded part, and after two or three weeks fits appeared, and the animal shortly afterwards had many very violent fits every day. I laid bare the sciatic nerve and cut it transversely. After this operation I kept the animal many months, and never saw it have a fit. From this fact, and from those observed by many physicians above related, it appears clearly that in cases of epilepsy it is necessary to examine if there is no injury whatever to some nerve, and more particularly when the *aura epileptica* exists. If there is such an injury, the treatment ought to be either the section of th injured nerve or the removal of the tumor, if there is one, and sometimes the application of a caustic or a blister.

XXVI.—LAWS OF THE DYNAMICAL ACTIONS IN MAN AND ANIMALS.*

The following laws are based upon a very considerable number of facts which I have observed or found on record in many books, pamphlets and journals. I have collected these facts and I intend to publish them in a special paper.

I ought to say that many Physiologists, and more particularly Fontana, Delaroche, Adamucci, Broussais, Buchez, Réveillé-Parise, J. Mueller, J. Paget and Carpenter, have pointed out the existence of some parts of some of these laws.

1. Nervous actions, muscular contraction, contraction of the cellular tissue, the discharge of the electrical apparatus of some fishes, the galvanic current of certain organs, the galvanic dis-

* I ought to say that these laws and the facts upon which they are established, have been the subject of many communications that I have made to the *Société de Biologie*, at Paris, in the year 1848.

charge which accompanies the muscular contraction, and *probably* also the phosphorescence of certain animals and the ciliary movements, are phenomena which cannot exist without being attended with an organic waste which nutrition alone can provide for.

2. The faculty of originating these phenomena has a tendency to increase in direct ratio to the rapidity of the circulation of blood, to its abundance, and to the amount of its nutritive materials, both general and special.

3. During rest, *i. e.* at the time of the non-existence of these phenomena, the tendency of such a faculty towards augmentation meeting with no obstacle, augmentation actually takes place.

4. The increase is much more rapidly effected when an action has just been performed, than it is after a prolonged rest.

5. Nutrition becoming altered in the tissues which remain inactive for a long time, the faculty of producing the above-mentioned phenomena diminishes by degrees, and even finally disappears when the structure of the tissues has been deeply modified.

6. The faculty of originating these phenomena increases in direct ratio with the length of the rest, within certain limits; and when the latter are overleaped, there is a period when no change takes place; but afterwards the faculty decreases, on the contrary, in direct ratio with the length of the rest.

7. For many tissues which produce these phenomena a complete rest is scarcely possible. The phenomena take place, in appearance, spontaneously, and with as much energy as the temperature is higher.

8. The faculty of producing these phenomena decreases at the time they are going on, in proportion to their intensity and duration, and in inverse ratio to the nutritive reparation which simultaneously takes place.

9. Reparation thus incessantly supplying for expenditure, it is not possible, with regard to most of these phenomena, and as long as circulation goes on, to destroy entirely the faculty of originating them; or rather, as soon as we have succeeded in destroying that faculty, it is reproduced by nutrition.

10. Expenditure dependent upon action, being followed by a great activity in the nutrition, it happens that, if the action be frequently renewed, there is an excess of nutrition and a considerable increase of the faculty of acting.

11. When the faculty of acting has been increased in virtue of the preceding reasons, within a certain limit, an equilibrium exists between the expenditure and the reparation, and the increase no longer takes place.

12. As it is possible for nutrition to take place in the tissues, although the nutritive fluid is not actually circulating in them, and provided that a certain amount of it exists in them, the faculty of acting may be increased in parts where circulation is stopped.

13. Although circulation and consequently reparation, are more active in summer than in winter time, at least in cold-blooded animals, the faculty of acting becomes more considerable in winter than in summer time, because the spontaneous expenditures abovementioned, and those due to external stimuli, or dependent upon the will, are by far less considerable.

All the preceding laws may be summed up in the following:

The intensity of the faculty which animal tissues possess, of producing the vital phenomena, seems to be in a direct ratio to the intensity and duration of the nutritive reparation, and in an inverse ratio to the intensity and duration of the existence of these phenomena.

